# Diploma Selling

**Published:** 1877-04

**Authors:** 


Diploma Selling.—We supposed the diploma trade could exist only in some big, bad city—Philadelphia, for example; but some facts that have just come to our knowledge show that this corruption can spread to the country, and establish its fountain of evil there. Arcadia, a pleasant village in Hamilton county, Indiana—Arcadia, the very name suggestive of Eden-like peace and purity!—it seems was the seat of a new tempter who was offering "diplomas from the best schools at home and abroad."

We publish *verbatim* one of the tempter's letters addressed to a reputable physician who, unfortunately for the success of the scheme, was a graduate. But at any rate one who could bite at such a bait would be sillier than the youngest minnow that ever sacrificed its life upon a crooked pin. And the doctor himself—the doctor so generous in offering diplomas to others—when and how did he get his diploma? However, it is well enough for the profession to l e warned in regard to this disreputable business. Here is the letter, written from Arcadia—written, too, on Christmas day! Place and day that should be sacred; is this not the climax of crime?

"Arcadia, Ind., Dec. 25th, 1876.

*Confidential Strictly:*

Dr. ———

Dear Sir: Under the above rule let me ask of you if you have ever obtained a Diploma, if not, would you like to obtain one.

If you are interested in this, come up and see me tomorrow.

Yours, truly,

J. G. D. Pettijohn.

Please do not mention this to any one."—*American Practitioner.*

